# The link between bone-derived factors osteocalcin, fibroblast growth factor 23, sclerostin, lipocalin 2 and tumor bone metastasis

**DOI:** 10.3389/fendo.2023.1113547

**Published:** 2023-02-28

**Authors:** Monika Martiniakova, Vladimira Mondockova, Roman Biro, Veronika Kovacova, Martina Babikova, Nina Zemanova, Sona Ciernikova, Radoslav Omelka

**Affiliations:** ^1^ Department of Zoology and Anthropology, Faculty of Natural Sciences and Informatics, Constantine the Philosopher University in Nitra, Nitra, Slovakia; ^2^ Department of Botany and Genetics, Faculty of Natural Sciences and Informatics, Constantine the Philosopher University in Nitra, Nitra, Slovakia; ^3^ Department of Genetics, Cancer Research Institute, Biomedical Research Center of Slovak Academy of Sciences, Bratislava, Slovakia

**Keywords:** osteocalcin, fibroblast growth factor 23, sclerostin, lipocalin 2, tumor bone metastasis

## Abstract

The skeleton is the third most common site of metastatic disease, which causes serious bone complications and short-term prognosis in cancer patients. Prostate and breast cancers are responsible for the majority of bone metastasis, resulting in osteolytic or osteoblastic lesions. The crosstalk between bone cells and their interactions with tumor cells are important in the development of lesions. Recently, both preclinical and clinical studies documented the clinical relevance of bone-derived factors, including osteocalcin (OC) and its undercarboxylated form (ucOC), fibroblast growth factor 23 (FGF23), sclerostin (SCL), and lipocalin 2 (LCN2) as prognostic tumor biomarkers and potential therapeutic targets in bone metastasis. Both OC and ucOC could be useful targets for the prevention of bone metastasis in breast cancer. Moreover, elevated OC level may be a metastatic marker of prostate cancer. FGF23 is particularly important for those forms of cancer that primarily affect bone and/or are characterized by bone metastasis. In other tumor entities, increased FGF23 level is enigmatic. SCL plays a significant role in the pathogenesis of both osteolytic and osteoblastic lesions, as its levels are high in metastatic breast and prostate cancers. Elevated expression levels of LCN2 have been found in aggressive subtypes of cancer. However, its role in anti-metastasis varies significantly between different cancer types. Anyway, all aforementioned bone-derived factors can be used as promising tumor biomarkers. As metastatic bone disease is generally not curable, targeting bone factors represents a new trend in the prevention of bone metastasis and patient care.

## Introduction

Tumor metastasis, the growth of primary tumor cells in a distant organ, involves cell migration, invasion, intra- and extravasation, survival in circulation, evasion in local immune responses, and eventual arrest at distant secondary sites (1, 2). The skeleton is considered the third most common site of metastatic disease after lung and liver. Prostate and breast cancers are responsible for most bone metastasis (up to 70%), although lung, kidney, thyroid and most adenocarcinoma primary tumors can metastasize to the bones as well (3, 4). The overall incidence of bone metastasis is not known. The relative incidence depends on the tumor type and in patients with advanced metastatic disease is: 65-75% in the prostate and breast, 60% in the thyroid, 30-40% in the lung and 20-25% in renal cell carcinoma (5). Bone metastasis influences survival rates ranging from 6 to 7 months for lung cancer to several years for breast cancer (19 to 25 months) or prostate cancer (12 to 53 months) and are a major cause of morbidity characterized by pathological fractures, spinal cord or nerve root compression, debilitating bone pain, bone marrow aplasia and hypercalcemia (2, 3).

Bone is a particularly suitable site for tumor cell metastasis mainly because it is a rich source of growth factors, neovascularization factors, and cytokines that facilitate the colonization, growth, and long-term survival of cancer cells (6). When cancer cells establish themselves in the bone microenvironment by disrupting bone homeostasis, this will result in increased bone resorption and/or bone formation. Overall, tumor cells interact with bone marrow cells and bone cells, thereby promoting tumor growth (7, 8). It is increasingly evident that osteoblasts in the bone microenvironment play a critical role in cancer cell attraction (9, 10), maintenance (11), and survival (6, 11, 12) during cancer progression in bone. The crosstalk between osteoblasts, osteocytes and osteoclasts is important in the development of bone metastasis (13).

**Figure 1:**
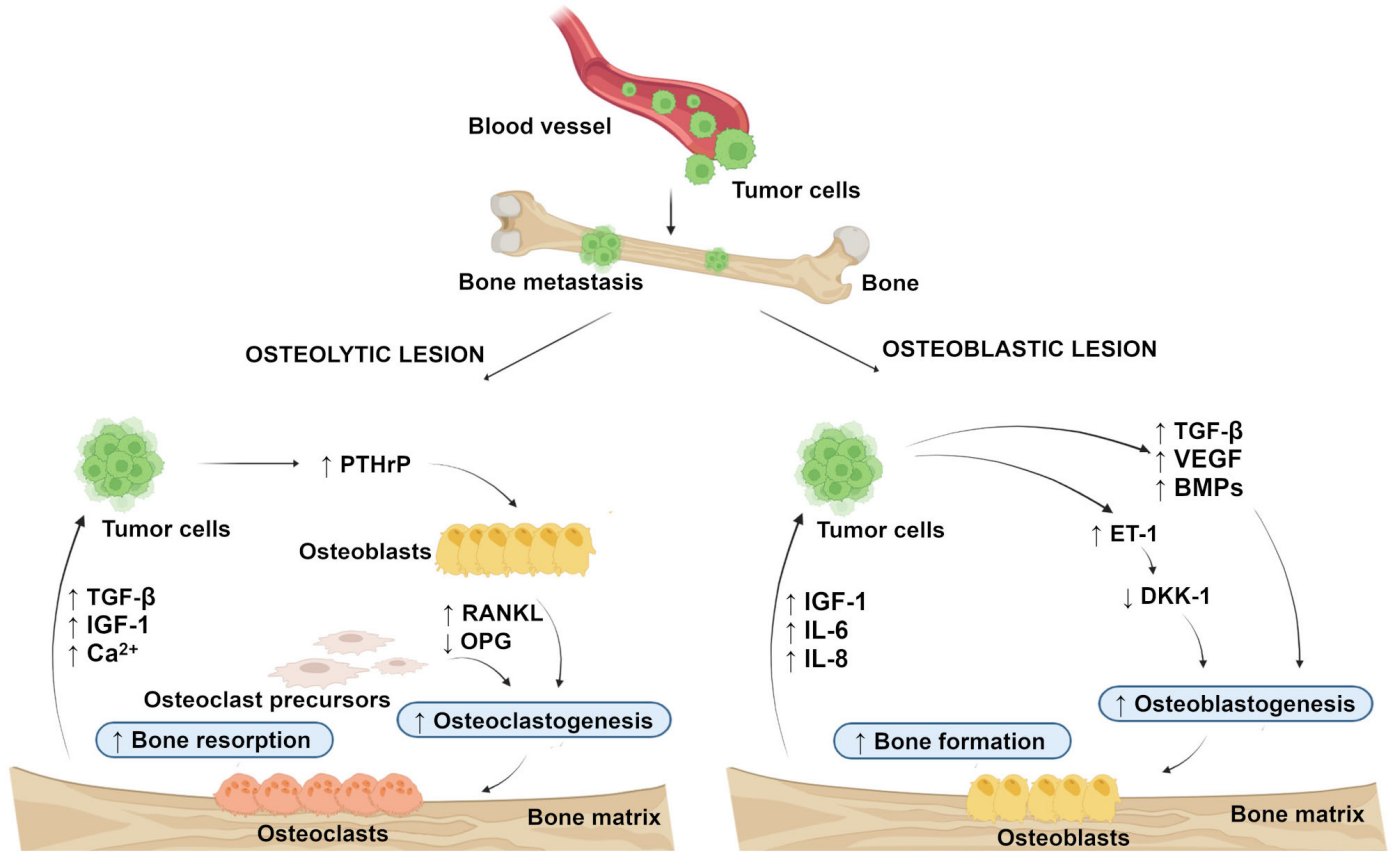
Schematic representation of the development of osteolytic and osteoblastic lesions. Osteolytic lesions are caused by excessive activation of bone resorption by osteoclasts in the tumor-bone microenvironment. Osteoblastic lesions are the result of direct tumor stimulation of osteoblasts, leading to uncontrolled bone formation. (Created with BioRender.com.) BMPs, bone morphogenetic proteins; DKK-1, Dickkopf-1; ET-1, endothelin-1; IGF-1, insulin-like growth factor-1; IL-6, interleukin-6; IL-8 interleukin-8; OPG, osteoprotegerin; PTHrP, parathyroid hormone-related peptide; RANKL, receptor activator of nuclear factor-kappa B ligand; TGF-β, transforming growth factor-beta; VEGF, vascular endothelial growth factor. ↑, increased; ↓, decreased.

In general, bone metastasis can be classified as osteolytic, osteoblastic, and mixed, according to the primary mechanism of interference with normal bone remodeling. Osteolytic metastasis is characterized by overactivation of bone resorption due to occurrence of osteoblast inactivation as well as the recruitment and activation of osteoclasts in the tumor-bone microenvironment (14, 15). Osteolytic lesions consist of soft parts of damaged bone with decreased bone mineral density (7). Parathyroid hormone-related peptide (PTHrP) plays a major role in their development (16). It is not clear whether the bone microenvironment induces cancer cells to express PTHrP, or if cells that metastasize to the bone have an intrinsic higher expression of PTHrP (17). The production of PTHrP up-regulates receptor activator of nuclear factor-kappa B ligand (RANKL) and down-regulates osteoprotegerin (OPG) by osteoblasts to activate osteoclastogenesis and bone resorption. Accelerated bone resorption, in turn, promotes the release of bone-derived growth factors such as transforming growth factor-beta (TGF-β), insulin-like growth factor (IGF)-1, and raises extracellular calcium concentration to further support the growth of cancer cells (**Figure 1**) (18, 19). In osteoblastic metastasis, on the other hand, the deposition of new bone occurs as a result of new bone formation (due to an osteoblastic response) that is not preceded by bone resorption (20). Osteoblastic lesions are the result of direct tumor stimulation of osteoblasts and are characterized by deposition of mineralized bone in tissue lesions (7). TGF- β, vascular endothelial growth factor (VEGF), bone morphogenetic proteins (BMPs) and endothelin (ET)-1 are associated with the generation of osteoblasts (21). ET-1 inhibits the expression of Dickkopf-1 (DKK-1), which normally blocks Wnt signaling. Inhibition of DKK-1 results in increased osteoblast activity supporting uncontrolled bone formation (22). Elevated bone formation drives tumor progression by releasing IGF-1 and interleukins (IL)-6 and IL-8 (**Figure 1**) (23). Finally, mixed bone metastasis involves a combination of both osteolytic and osteoblastic components (2).

Osteocalcin (OC), fibroblast growth factor 23 (FGF23), sclerostin (SCL), and lipocalin 2 (LCN2) are bone-derived factors secreted by bone cells that are involved in the regulation of bone homeostasis. Their implication in cancer biology has attracted research interest in recent years. This review aims to summarize the current knowledge about OC, FGF23, SCL, and LCN2 and to characterize the association between them and tumor bone metastasis.

## Osteocalcin and tumor bone metastasis

OC is the most abundant non-collagenous protein in bone. It is secreted by osteoblasts and contains three glutamate residues that can be carboxylated, which then provide high affinity to the hydroxyapatite matrix. This modification is catalyzed by γ-glutamyl carboxylase, and uses vitamin K, O_2_, and CO_2_ as cofactors, supplied by the vitamin K cycle and circulation (24, 25). Carboxylated OC (cOC) is essential for the alignment of apatite crystals and optimal bone strength (26). Undercarboxylated OC (ucOC) with a reduced degree of carboxylation at the glutamate residues is available with less affinity for hydroxyapatite (25). When bone is resorbed by osteoclasts, the acidic pH in the resorption lacuna causes the carboxyl groups on OC to be removed and ucOC to be released into the systemic circulation. Therefore, circulating levels of ucOC are dependent on the rate of bone remodeling (27, 28). Generally, ucOC is considered an endocrinologically active form that can regulate glucose metabolism, testosterone synthesis, muscle mass, and cognitive function (29–32).

In addition, the relationship between OC and tumorigenesis has been noted (33). Pietschmann et al. (34) and Salem et al. (35) indicated significantly increased serum OC levels in breast cancer patients and subjects with bone metastasis compared to healthy controls (35). The research by Xu et al. (36) showed that ucOC facilitated the proliferation and metastasis of breast cancer cells MDA-MB-231 by accelerating the TGF-β/SMAD3 signaling pathway. Furthermore, ucOC also promoted the gene expression of IL-8 and PTHrP, which may act as osteolytic factors in breast cancer cells. Therefore, ucOC could be a useful target for the prevention of bone metastasis in breast cancer. A tumor-promoting effect of OC on human prostate cancer cell line PC-3 and pancreatic cancer cells was revealed by Ye (37) and Kayed et al. (38). OC has also been found to facilitate the growth and migration of lung tumors through neutrophil aggregation (39) and to induce the growth of prostate cancer cells *via* G protein-coupled receptor family C group 6 member A (GPRC6A) receptor (40). OC production correlates with prostate cancer metastasis (41). Therefore, high serum OC level may be a metastatic marker of prostate cancer (42). Relevant information related to this issue is presented in **Table 1**. The aforementioned studies provide an interesting basis for further research regarding the roles of OC and ucOC in tumorigenesis.

**Table 1:** Associations of OC, FGF23, SCL, and LCN2 with cancer.

Factor	Cancer type	Factor level	Factor effect/association	References
OC	Breast cancer	↑serum	↑bone metastasis ↑bone plus soft tissue metastasis	Salem et al., 2007 (35) ; Pietschman et al., 1989 (34)
	Breast cancer metastatic cells	ucOC treatment	↑proliferation and metastasis ↑TGF-β/SMAD3 signaling pathway ↑gene expression of IL-8 and PTHrP	Xu et al., 2022 (36)
	Prostate cancer cell line PC-3, and PC-3 injected into mice	OC treatment	Activation of GPRC6A ↑PC-3 cell number and migration ↑ERK, Akt, and mTOR phosphorylation	Ye et al., 2017 (37)
	Prostate cancer cell lines	OC treatment	Activation of GPRC6A ↑ERK activity ↑cell proliferation ↑chemotaxis	Pi and Quarles, 2012 (40)
	Prostate cancer samples	↑bone-metastasized tumors	Not determined	Gardner et al., 2009 (41)
	Prostate cancer	↑serum	↑advanced-stage prostate cancer	Nimptsch et al., 2009 (41)
FGF23	Bone metastases from solid tumors	↑serum	↓overall survival and time to skeletal-related events	Mansinho et al., 2019 (43)
	Myelodysplastic syndromes mouse model	↑serum	Delays bone mineralization ↑number of osteoblasts and fraction of non-mineralized bone ↓trabecular number	Weidner et al., 2020 (44)
	Multiple myeloma and cell lines	↑serum	↑αKlotho expression in myeloma cells ↑EGR1 and HPSE	Suvannasankha et al., 2015 (45)
	Prostate cancer	↑serum	↑aggressive behavior of prostate cancer ↑FGF23 expression via a positive autocrine feedback loop	Lee et al., 2014 (46)
	Prostate cancer samples and cell lines	↑cells	↑cell proliferation and tumor invasion	Feng et al., 2015 (47)
	Prostate cancer	↔plasma	Not determined	Vlot et al., 2018 (48)
	Prostate cancer ex vivo model	↑osteocytes	Not determined	Choudhary et al., 2018 (49)
	Tumor-induced osteomalacia	↑serum	↓1,25-dihydroxyvitamin D ↓phosphate ↑ALP	Cotant and Rao, 2007 (50); Chiam et al., 2013 (51)
	Oncogenic osteomalacia	↑serum	↓phosphate ↑ALP	Savva et al., 2019 (52); Abramson et al., 2021 (53)
SCL	Multiple myeloma	↑bone marrow	↑MIP-1α, β-2 microglobulin and Ca levels ↓bALP levels ↓median survival	Wang et al., 2014 (54)
	Multiple myeloma and samples	↑plasma	↓β-catenin expression ↓bALP ↑CTx ↓median survival	Eda et al., 2016 (55); Terpos et al., 2012 (56)
	Multiple myeloma samples and mice model	↑osteocytes	↑bone loss ↓fracture resistance	McDonald et al., 2017 (57)
	Breast cancer cell lines	↑cells	Mediator of Runx2/CBFβ action in metastatic cells ↓osteoblast differentiation	Mendoza-Villanueva et al., 2011 (58)
	Prostate cancer specimens	↓cells	↑prostate cancer metastases (in combination with BMP-6 and noggin)	Yuen et al., 2008 (59)
	Prostate cancer metastatic to the skeleton	↑serum	↑bone turnover ↑P1NP	Yavropoulou et al., 2012 (60)
	Prostate cancer	↑serum	↓testosterone	Garciá-Fontana et al., 2014 (61)
LCN2	Breast cancer cell lines	↑cells	↑cell motility and invasiveness	Yang et al., 2009 (62)
	Breast cancer samples	↑cells	↓estrogen receptor, progesterone receptor status ↑presence of lymph node metastases ↓disease-specific survival	Bauer et al., 2008 (63)
	Pancreatic cancer samples	↑cells	Not determined	Moniaux et al., 2008 (64)
	Thyroid carcinoma cell line	↑cells	↑malignant phenotype of tumors ↑NF-κB basal activity	Iannetti et al., 2008 (65)
	Colorectal cancer specimens	↑cells	Not determined	Nielsen et al., 1996 (66)
	Prostate cancer specimens	↑cells	↑migration of prostate cancer cells ↑tumor metastasis ↑Src signaling	Lu et al., 2019 (67)

ALP, alkaline phosphatase; bALP, bone-specific alkaline phosphatase; BMP-6, bone morphogenetic protein 6; CBFβ, core binding factor beta; CTx, C-terminal telopeptide of type I collagen; EGR1, early growth response factor 1; ERK, extracellular signal-regulated kinase; FGF23, fibroblast growth factor 23; GPRC6A, G protein-coupled receptor family C group 6 member A; HPSE, heparanase; IL-8, interleukin-8; LCN2, lipocalin 2; MIP-1α, macrophage inflammatory protein-1 alpha; mTOR, mammalian target of rapamycin; NF-κB, nuclear factor kappa B; OC, osteocalcin; P1NP, N-terminal propeptide of type 1 collagen; PTHrP, parathyroid hormone-related peptide; Runx2, runt-related transcription factor-2; SCL, sclerostin; SMAD3, SMAD Family Member 3; Src, steroid receptor coactivator; TGF-β, transforming growth factor-beta; ucOC, undercarboxylated osteocalcin. ↑, increased; ↓, decreased.

## Fibroblast growth factor 23 and tumor bone metastasis

FGF23 is a bone-derived protein predominantly secreted by osteoblasts and osteocytes (68). Its target organs include the kidney and parathyroid glands (69). In the kidney, FGF23 acts as a hormone stimulating phosphate excretion and suppressing the synthesis of 1.25(OH)_2_D_3_, an active vitamin D. In the parathyroid glands, FGF23 reduces the production and secretion of parathyroid hormone (PTH). These endocrine effects are dependent on the transmembrane protein αKlotho, which increases the binding affinity of FGF23 to FGF receptors (FGFR) (70). In this way, FGF23 is part of a hormonal circuit that additionally involves PTH and 1.25(OH)_2_D_3_ and regulates phosphate and vitamin D metabolism, as well as affecting Ca^2+^ (71). Locally produced FGF23 in other tissues, including the liver or heart, has additional paracrine effects without the involvement of αKlotho (70). Osteoblasts, osteocytes, and osteoclasts express the FGFR/αKlotho coreceptor. The specific knockout of αKlotho in osteocytes can lead to the osteogenic enhancement and increased bone mass (72). Several studies have also shown that FGF23 can upregulate early growth response genes in osteoblasts and the RANKL/OPG ratio in the osteoclast surface by binding to the coreceptor. Regardless, FGFR/αKlotho coreceptor activation may also account for bone remodeling (73).

In those forms of cancer that involve or originate from bone, FGF23 signaling may directly contribute to cancer biology and/or progression (70). In patients with cancer and bone metastases from solid tumors, elevated serum levels of FGF23 were associated with shorter survival, as well as time to skeletal-related events (43), providing the first evidence of the prognostic significance of FGF23 in individuals with bone metastasis. Mouse model of myelodysplastic syndrome also had increased level of FGF23 (44). Serum levels of intact FGF23 were found to be higher also in subjects with multiple myeloma (45). FGF23 expression was increased in patients with prostate cancer, as well as FGF23/FGFR1/αKlotho in various prostate cancer cell lines (46). For this aim, FGF23 can act as an autocrine factor in prostate cancer cells stimulating tumor invasion and cell proliferation (47). According to Vlot et al. (48), the serum level of FGF23 was unchanged in prostate cancer (48), although prostate cancer cells can stimulate FGF23 expression in osteocytes (49). However, bone metastasis may account for the high FGF23 levels and symptoms of tumor-induced osteomalacia identified in prostate cancer patients (50, 51). Breast cancer may also be consistent with oncogenic osteomalacia and elevated levels of FGF23 (52, 53). In all of these reported cases, including patients with bone metastases, increased concentration of FGF23 was accompanied by a decrease in the serum phosphate level. FGF23 mRNA expression was high in breast cancer cells as well (74). In addition, FGFR signaling may be highly relevant to breast cancer oncogenesis (75). **Table 1** summarizes relevant studies reporting FGF23 levels and its function in several cancer types. An anti-FGF23 approach may be useful in malignancies affecting bone because they are characterized by enhanced FGF23 levels or FGF23 signaling. In other tumor entities (e.g. endometrial cancer, hepatocellular carcinoma), no change in serum FGF23 level was demonstrated (70). It is important to note that most of the above-mentioned studies report associations, not necessarily causal relationships, but FGF23 may serve as a tumor biomarker.

## Sclerostin and tumor bone metastasis

SCL is a glycoprotein encoded by the SOST gene and secreted mainly by osteocytes but can be widely expressed in other tissues and organs, such as the kidney, liver, and lung (76, 77). SCL expression depends on hormones, inflammatory molecules, and mechanical loading (78). SCL can suppress the activity of osteoblasts and osteoclasts, which stabilizes bone strength and toughness under normal physiological conditions (79). Lack of SCL will lead to excessive bone hardening and its overexpression will inhibit bone formation (80). SCL binds to lipoprotein receptor-related proteins (LRP) 5/6, critical coreceptors of the Wnt signaling pathway, leading to decreased bone formation (76, 81). Since the canonical Wnt signaling pathway is a key regulator of cellular functions including proliferation, differentiation and migration in various tissues, an endocrine role of SCL has been identified as well (82, 83). Moreover, the aforementioned pathway is also involved in the pathogenesis of many skeletal disorders and cancer (77).

In patients with multiple myeloma, serum and bone marrow SCL levels were elevated (54, 55) and increased serum SCL correlated with poor survival (56). Genetic deletion of the SOST gene was found to reduce osteolytic lesions in immunodeficient mice by increasing and decreasing the number of osteoblasts and osteoclasts in multiple myeloma (84). Similarly, pharmacological inhibition of SCL with an anti-SCL antibody in immunocompetent mice decreased osteolysis and elevated markers of bone formation without altering tumor growth (57). SCL was elevated in both metastatic breast cancer (MDA-MB 231) and non-metastatic (MCF-7) cell lines, due to abnormal overexpression of runt-related transcription factor-2 (Runx2), which binds to the proximal promoter of the SOST gene. Increased levels of SCL in metastatic breast cancer suppressed bone formation through inhibition of the Wnt signaling pathway, suggesting its role in the pathogenesis of osteolytic lesions (58). Yuen et al. (59) found that high expression levels of BMP-6 and low expression levels of its inhibitors SCL and noggin in primary prostate cancer significantly predicted the development of distant metastasis. SCL was found to inhibit prostate cancer migration and its deficiency led to the increased spread of prostate cancer (85). Yavropoulou et al. (60) reported a high correlation between serum SCL levels and elevated bone turnover. The authors explained this finding by a compensatory response to the increased number of osteoblasts at affected skeletal sites which may contribute to enhanced bone resorption. Elevated levels of circulating SCL have also been found in prostate cancer patients receiving androgen deprivation therapy (61). The inverse relationship between serum SCL and testosterone in these patients points to an important influence of androgens in the regulation of bone metabolism in prostate cancer. All these findings suggest that SCL could have a protective role in prostate cancer progression, and elevated serum SCL levels reflect the increased activity of osteoblasts and osteocytes in prostate cancer-induced osteoblastic lesions. Findings reporting SCL levels and its function in several types of cancer are shown in **Table 1**.

## Lipocalin 2 and tumor bone metastasis

LCN2, also known as neutrophil gelatinase-associated lipocalin or 24p3, is a secreted glycoprotein associated with many functions such as stimulation of neutrophil migration (86), regulation of bone homeostasis (87) and skeletal muscle regeneration (88). LCN 2 was previously understood as one of the adipokines (89); however, its expression level was recently found to be at least 10-fold higher in osteoblasts compared to adipocytes. In mice with a conditional knockout of LCN2 in osteoblasts, elevated levels of plasma glucose and body fat were reported (76, 90). In bone, LCN2 is regulated by physical activity/inactivity (87) and negatively modulates bone development and turnover, as overexpression of LCN2 results in thinner cortical bone and reduced osteoblast differentiation (91). Recently, bone-derived LCN2 has been shown to have endocrine functions in appetite control and insulin secretion. According to Mosialou et al. (90), LCN2 binds to melanocortin receptor 4 (MC4R) of the paraventricular neurons in the hypothalamus and directly suppresses appetite. Moreover, the administration of LCN2 has been shown to improve glucose metabolism and energy expenditure in a mouse model (90). In patients with type 2 diabetes, higher serum LCN2 levels were associated with obesity, dyslipidemia, and insulin resistance. Therefore, LCN2 could serve as a biomarker for metabolic diseases (92). Under pathological conditions, LCN2 expression can be induced in a wide range of organs, including the kidney (93) and liver (94).

Expression levels of LCN2 have been found to be high in aggressive subtypes of cancer, including breast, pancreas, thyroid, and colon cancers (62, 64–66). In general, LCN2 may promote tumorigenesis by enhancing invasion, proliferation, and metastasis while reducing apoptosis. Some of these characteristics arise from the ability of LCN2 to facilitate iron uptake into cancer cells or its ability to form a heterodimer with matrix metalloprotease (MMP)-9, which contributes to tumor progression and metastasis (95). Studies by Yang et al. (62) and Bauer et al. (63) confirmed the presence of LCN2 in invasive breast cancer tissues and cell lines. LCN2 levels were increased in advanced stages of cancer and they were associated with decreased overall survival. Moniaux et al. (64) revealed higher LCN2 expression in neoplastic lesions of pancreatic tissue that typically develop into pancreatic cancer. In pancreatic cancer tissues, LCN2 expression was upregulated compared to tissues from healthy individuals (64, 96). According to Iannetti et al. (65), thyroid carcinoma-derived tissues also had higher LCN2 expression levels versus normal thyroid tissue. Increased expression of LCN2 in colon tumor tissues was found by Nielsen et al. (66). However, no significant differences in LCN2 expression between neoplastic and non-neoplastic tissues were noted. In metastasis, LCN2 was initially identified as a promoter to induce epithelial–mesenchymal transition (EMT) in breast cancer cells to promote tumor metastasis (62). In prostate cancer cells, LCN2 played an important role in facilitating cell migration and invasion by inducing EMT, leading to increased tumor invasion (67). However, disruption of the LCN2 gene suppressed primary mammary tumor formation in mice, while not reducing lung metastases (97). Thus, the role of LCN2 in metastasis may differ significantly between different cancer types. However, LCN2 could be a prognostic biomarker for this disease. In some types of cancer (especially breast cancer), the development of therapeutic agents targeting LCN2 may have major clinical implications for the treatment of metastasis (98). **Table 1** summarizes relevant information related to this issue.

## Conclusion

Metastatic bone disease leads to a significant decrease in quality of life and is strongly correlated with high morbidity and poor outcomes in cancer patients. Increasing evidence identified the bone marrow as a preferred metastatic site for several solid neoplasms, including breast, prostate, lung, and kidney tumors. Osteoblasts, osteocytes, and osteoclasts represent key regulators of bonehomeostasis, and their interactions with tumor cells are considered critical for metastatic colonization in the bone marrow. However, their role in remodeling the bone microenvironment, leading to a supportive niche for tumor cell proliferation and survival, needs to be evaluated. Findings from cell cultures, animal studies, and clinical trials have recently documented the importance of various bone-derived factors as prognostic biomarkers and potential therapeutic targets in bone metastasis. Large clinical studies involving metastatic/non-metastatic patients and non-cancer control subjects may bring crucial findings for more efficient therapies to combat metastasis. Further research aimed at better understanding the tumor-specific pathways associated with bone metastasis may identify potential tumor-specific therapeutic targets.

## Author contributions

Conceptualization, MM and RO. Methodology, MM, VM and RO. Formal analysis, RB, VK, MB and NZ. Writing-original draft preparation, MM, VM, SC and RO. Writing-review and editing, MM, SC and RO. Visualization, RB, VK, MB and NZ. Supervision, MM and RO. Funding acquisition, RO. All authors contributed to the article and approved the submitted version.

## Glossary

**Table:**

bALP	bone-specific alkaline phosphatase
BMP-6	bone morphogenetic protein 6
BMPs	bone morphogenetic proteins
CBFβ	core binding factor beta
cOC	carboxylated osteocalcin
CTx	C-terminal telopeptide of type I collagen
DKK-1	Dickkopf-1
EGR1	early growth response factor 1
EMT	epithelial–mesenchymal transition
ERK	extracellular signal-regulated kinase
ET-1	endothelin-1
FGF23	fibroblast growth factor 23
FGFR	fibroblast growth factor receptor
GPRC6A	G protein-coupled receptor family C group 6 member A
HPSE	heparanase
IGF-1	insulin-like growth factor-1
IL-6	interleukin-6
IL-8	interleukin-8
LCN2	lipocalin 2
LRP	lipoprotein receptor-related proteins
MC4R	melanocortin receptor 4
MIP-1α	macrophage inflammatory protein-1 alpha
MMP	matrix metalloprotease
mTOR	mammalian target of rapamycin
NF-κB	nuclear factor kappa B
OC	osteocalcin
OPG	osteoprotegerin
P1NP	N-terminal propeptide of type 1 collagen
PTH	parathyroid hormone
PTHrP	parathyroid hormone-related peptide
RANKL	receptor activator of nuclear factor-kappa B ligand
Runx2	runt-related transcription factor 2
SCL	sclerostin
SMAD3	SMAD Family Member 3
Src	steroid receptor coactivator
TGF-β	transforming growth factor-beta
ucOC	undercarboxylated osteocalcin
VEGF	vascular endothelial growth factor
